# Ceftobiprole: a potential empirical post-operative monotherapy in prosthetic joint infections

**DOI:** 10.1186/s12941-020-00351-5

**Published:** 2020-03-21

**Authors:** Claire Duployez, Frédéric Wallet, Henri Migaud, Eric Senneville, Caroline Loiez

**Affiliations:** 1 Institute of Microbiology, Lille University Hospital, 59037 Lille, France; 2 Orthopaedic Department, Lille University Hospital, 59037 Lille, France; 3 Infectious Diseases Department, Gustave Dron Hospital, 59200 Tourcoing, France; 4 University Hospital of Lille, 59037 Lille, France

**Keywords:** Prosthetic joint infection, Ceftobiprole, Empirical antibiotic therapy, Bacterial resistance

## Abstract

**Background:**

This study aimed to evaluate in vitro susceptibility to ceftobiprole of clinical strains identified from prosthetic joint infections (PJIs) compared to that of the associations currently recommended for post-operative empirical antibiotic therapy (PEAT) (vancomycin with either cefepime, third-generation cephalosporin or piperacillin–tazobactam).

**Methods:**

We performed a 1-year retrospective study on all the surgical procedures performed in our hospital for PJI. Susceptibility profiles of all the strains cultured from surgical samples were reviewed to compare ceftobiprole to current used associations.

**Results:**

During the study period (from January 2018 to December 2018), we identified 106 patients managed for PJI and a total of 216 surgical interventions. One hundred-fifty strains were identified from intraoperative samples, excluding redundant strains. *Staphylococcus* spp. represented 52.7% of all strains and Enterobacteriales 13.3%. Twenty-three patients had polymicrobial infection (22%). Among 149 surgical procedures with positive culture results, ceftobiprole covered the bacterial strains in 138 (92.6%) cases. In comparison, this percentage was 94.6% for vancomycin plus cefepime (p = 0.64), 92.6% for vancomycin plus a third-generation cephalosporin in 138 cases (p = 1) and 94.6% for vancomycin plus piperacillin–tazobactam) (p = 0.64).

**Conclusion:**

Based on antimicrobial susceptibility testing, our results suggest that ceftobiprole could be an interesting option for PEAT in PJIs, allowing the use of a single agent.

## Background

Prosthetic joint infections (PJI) require both surgical intervention and antibiotic therapy conducted according to the most recent guidelines for the management of these potentially life-threatening infections [1, 2].

The results of the susceptibility profile of the bacteria isolated from the intraoperative samples usually require at least 5 days to become available. During this period, an initial post-operative empirical antibiotic therapy (PEAT) is usually administered to prevent the colonization of newly prosthesis or the prosthesis that has been cleaned but has been retained during the so-called debridement-antibiotic and implant retention (DAIR). Given the important role of staphylococci in PJI, the PEAT needs to be active against most Gram-positive cocci including methicillin-resistant staphylococci but also Gram-negative bacilli. Currently, there has been no consensus on the optimal choice of this antibiotic therapy. In France, the combinations of vancomycin combined with either a third-generation cephalosporin or piperacillin–tazobactam are proposed choices [3].

Ceftobiprole is a newly commercialized beta-lactam antibiotic with a broad spectrum, equivalent to an association of a third-generation cephalosporin plus vancomycin. Notably, it is active against methicillin-resistant staphylococci, *Enterococcus faecalis*, most extended spectrum beta-lactamase-non-producing Enterobacteriales and *Pseudomonas aeruginosa*. It may replace use of this associations for PEAT.

The main objective of the present study was to assess the antibacterial in vitro activity of ceftobiprole on strains recovered from PJI infections in order to enable or not its use as a single molecule in PEAT. We therefore compared ceftobiprole to vancomycin plus cefepime, vancomycin plus third generation cephalosporin (e.g. cefotaxime or ceftriaxone) and vancomycin plus piperacillin–tazobactam in this setting.

## Methods

### Definitions

PJIs were identified according to the Infectious Diseases Society of America definition [2].

### Study design and population

This retrospective study was performed at the French National Reference Centre for Complex Osteoarticular Infections in the North West region of France (Roger Salengro Hospital, Lille, France). Medical charts of all adult patients with documented PJI who received PEAT from January 2018 to December 2018 were reviewed. All patients included in the study had surgical management including DAIR, one or two-step implant exchange.

### Surgical management and curative antibiotic therapy

All surgical procedures (i.e., implant retention, or one- to two-step implant exchange) were performed without antibiotic prophylaxis. Ceftobiprole administered intravenously was begun intraoperatively immediately after samples were taken. PEAT was continued until the results of intraoperative sample cultures were available and was then modified in accordance with the culture results (curative antibiotic therapy). Therapeutic strategies were decided for each patient during multidisciplinary meetings of orthopaedic surgeons, infectious diseases consultants, microbiologists and anaesthesiologists, based on the patient’s characteristics and were administered following the recommendations of Zimmerli et al. [4]. In each case, the patient was aware of the different therapeutic options and took part in the final decision.

### Microbiology

During surgical procedures, at least three tissue samples were taken in different areas suspected of being affected using a separate sterile instrument for each sample. Three mL of sterile saline were added to each sample, and samples were vigorously shaked during 1 min using sterile glass beads, in order to disrupt tissue and release bacteria. Then, one drop was inoculated onto a Columbia agar with blood 5% (incubation at 37 °C in air for 5 days), one drop onto a chocolate agar with polyvitex (incubation at 37 °C in CO_2_ for 5 days) and one-millilitre aliquots were inoculated into an Aerobic VIRTUO blood culture bottle, and an anaerobic VIRTUO blood culture bottle (BioMérieux, Marcy l’Etoile, France). All plates were examined daily for 5 days. VIRTUO blood culture bottles were placed on the VIRTUO system for 14 days and were subcultured if they flagged positive.” Strains were identified using MALDI-TOF spectrometry mass (Bruker Daltonics, Wissembourg, France) with a minimal score requirement of 2.

The antibiotic susceptibility profile of all pathogens identified from intraoperative samples was assessed either by the Vitek 2 cards (BioMérieux, Marcy l’Etoile, France) or by agar diffusion technique using the procedure and interpretation criteria proposed by the Comité de l’Antibiogramme de la Société Française de Microbiologie (CA-SFM EUCAST 2018) (http://www.sfm-microbiologie.org). Minimum inhibitory concentrations (MIC) of ceftobiprole were determined with the Etest (Liofilchem, Roseto degli Abruzzi, Italy). Methicillin resistance was confirmed by detection of *mecA* gene if required.

## Results

### Patients

During the study period, we identified 106 patients managed for PJI (57 hip prosthesis, 39 knee prosthesis, 9 shoulder prosthesis, 4 elbow prosthesis and 4 other prosthesis, with sometimes more than one prosthesis by patient). A total of 216 surgical interventions were identified from these 106 patients. Among these 216 surgical interventions, 67 remained negative for the three samples (31%).The demographic characteristics of the included patients are reported in Table 1.

**Table 1:** Characteristics of 106 patients (216 events) with PJI

Characteristics	
Male, N of patients (%)	53 (50)
Female, N of patients (%)	53 (50)
Age, years, mean ± SD (range)	67 ± 12.9 (26–95)
≥ 75 (%)	27 (25.5)
50–75 (%)	70 (66)
< 50 (%)	9 (8.5)
Location of PJI, N of patients (%)	
Hip	57
Knee	39
Shoulder	9
Elbow	4
Other prosthesis	4
Fever at the time of surgical management, N of events (%) (data available for 214 events)	14 (6.5%)
Biological characteristics at the time of surgical management	
CRP (mg/L) ± SD (range) (data available for 164 events)	76.64 ± 91.9 (< 3–431)
Leucocytes (×10^9^/L) ± SD (range) (data available for 177 events)	9.35 ± 3.51 (4.30–29.42)
Creatinine (mg/L) ± SD (range) (data available for 178 events)	8.60 ± 3.76 (3–29)

*SD* standard deviation

### Microbiology

A total of 150 clinical strains were identified from intraoperative samples, excluding redundant strains taken in different surgical interventions performed on the same patient (Table 2).

**Table 2:** Pathogens isolated from intraoperative samples of PJI in 106 patients and their susceptibility profile to the antimicrobial therapy evaluated

Microorganism	Number of strains (% of the total)	Ceftobiprole: resistant strains	Vancomycine + cefepime: resistant strains	Vancomycine + 3rd generation cephalosporin: resistant strains	Vancomycine + piperacillin–tazobactam: resistant strains
Gram-positive cocci	111 (74.0)	1	0	0	0
Staphylococci	79 (52.7)	–	–	–	–
*Staphylococcus aureus*	30 (20.0)	–	–	–	–
MSSA	28 (18.7)	–	–	–	–
MRSA	2 (1.3)	–	–	–	–
CoNS	49 (32.7)	–	–	–	–
*Staphylococcus epidermidis*	30 (20.0)	–	–	–	–
*Staphylococcus capitis*	7 (4.7)	–	–	–	–
*Staphylococcus caprae*	4 (2.7)	–	–	–	–
*Staphylococcus haemolyticus*	1 (0.7)	–	–	–	–
*Staphylococcus lugdunensis*	4 (2.7)	–	–	–	–
*Staphylococcus pettenkoferi*	2 (1.3)	–	–	–	–
*Staphylococcus warneri*	1 (0.7)	–	–	–	–
Streptococcus spp.	20 (13.3)	–	–	–	–
*Streptococcus adiacens*	1 (0.7)	–	–	–	–
*Streptococcus anginosus*	1 (0.7)	–	–	–	–
*Streptococcus agalactiae*	6 (4.0)	–	–	–	–
*Streptococcus dysgalactiae*	3 (2.0)	–	–	–	–
*Streptococcus gallolyticus*	1 (0.7)	–	–	–	–
*Streptococcus gordonii*	2 (1.3)	–	–	–	–
*Streptococcus mitis/oralis*	2 (1.3)	–	–	–	–
*Streptococcus pneumoniae*	1 (0.7)	–	–	–	–
*Streptococcus parasanguinis*	1 (0.7)	–	–	–	–
*Streptococcus sanguinis*	1 (0.7)	–	–	–	–
*Streptococcus vestibularis*	1 (0.7)	–	–	–	–
Enterococci	12 (8.0)	1	–	–	–
*Enterococcus faecalis*	11 (7.3)	–	–	–	–
*Enterococcus raffinosus*	1 (0.7)	1	–	–	–
Gram-negative bacilli	24 (16.0)	6	5	8	4
Enterobacteriales	20 (13.3)	4	4	4	3
*Citrobacter freundii*	1 (0.7)	–	–	–	–
*Citrobacter koseri*	1 (0.7)	–	–	–	–
*Enterobacter cloacae (2 ESBL*-*producing strain)*	3 (2.0)	2	2	2	2
*Escherichia coli (1 ESBL*-*producing strain)*	4 (2.7)	1	1	1	1
*Klebsiella oxytoca*	1 (0.7)	–	–	–	–
*Klebsiella pneumoniae (1 ESBL*-*producing strain)*	4 (2.7)	1	1	1	2
*Morganella morganii*	1 (0.7)	–	–	–	–
*Proteus mirabilis*	4 (2.7)	–	–	–	–
*Serratia marcescens*	1 (0.7)	–	–	–	–
Non-fermenting bacilli	4 (2.7)	2	1	4	1
*Pseudomonas aeruginosa*	3 (2.0)	1	–	3	–
*Acinetobacter baumannii (OXA23*-*producing strain)*	1 (0.7)	1	1	1	1
Anaerobes	8 (5.3)	1	1	1	0
*Cutibacterium acnes*	5 (3.3)	–	–	–	–
*Cutibacterium avidum*	1 (0.7)	–	–	–	–
*Clostridium perfringens*	1 (0.7)	–	–	–	–
*Bacteroides thetaiotaomicron*	1 (0.7)	1	1	1	–
Others	7 (4.7)	2	2	2	2
*Bacillus cereus*	1 (0.7)	–	–	–	–
*Bacillus pumilus*	1 (0.7)	–	–	–	–
*Corynebacterium striatum*	1 (0.7)	–	–	–	–
*Micrococcus luteus*	2 (1.3)	–	–	–	–
Yeast	2 (1.3)	2	2	2	2
Total number of strains	150 (100)	10	8	11	6

*Staphylococcus* spp. accounted for 52.7% of all strains, especially coagulase negative staphylococci (CoNS) (32.7% of all strains) with 53.1% of methicillin-resistant and 20.4% of teicoplanin-resistant strains. *S. aureus* accounted for 20.0% of all strains including 2 methicillin-resistant *S. aureus* (MRSA). *Streptococcus* spp. and *Enterococcus* spp. accounted for 21.3% of all strains. Enterobacteriales represented 13.3% of all strains including 4 extended-spectrum beta-lactamases (ESBL)-producing Enterobacteriales. Among non-fermenting Gram-negative bacilli, 3 strains of *Pseudomonas aeruginosa* and 1 strain of *Acinetobacter baumannii* were identified. At last, for 2 patients, yeasts were identified. Twenty-three patients had polymicrobial infection (22%).

We evaluated the in vitro susceptibility to ceftobiprole, and to the associations of vancomycin with either cefepime, a third-generation cephalosporin (cefotaxime, ceftriaxone) or piperacillin–tazobactam. Results are presented in Table 2 and resistant strains to at least one of these therapies are detailed in Table 3. Among Gram-positive cocci, all strains were susceptible to the 3 associations and all but the strain of *E. raffinosus* were susceptible to ceftobiprole. Overall, 100% of *S. aureus* and CoNS strains were inhibited at the CASFM EUCAST breakpoints of 2 mg/L (breakpoint for *S. aureus*) and 4 mg/L (non-species-related breakpoint), respectively. (MIC_50/90_ of 0.5/0.75 and 0.5/1 respectively). Only one methicillin-resistant *S. haemolyticus* isolate had a MIC of 4 mg/L. For Enterobacteriales, 4 ESBL-producing strains were resistant to ceftobiprole as to the association including cefepime or a third-generation cephalosporin (patients C, D, G, H). Piperacillin–tazobactam was effective against one of these ESBL-producing strains (patient C). The 3 *P. aeruginosa* strains were susceptible to cefepime and piperacillin–tazobactam; one was resistant to ceftobiprole. OXA23-producing *A. baumannii* was resistant to the 4 therapies evaluated. Due to their spectrum of activity, none of these therapies was effective on the 2 yeasts and none except piperacillin–tazobactam was effective on *B.* *thetaiotaomicron*.

**Table 3:** Susceptibility profile of the 16 strains resistant to at least 1 of the antimicrobial therapy evaluated

Patient	Surgical procedure	Strains	Ceftobiprole	Vancomycin–cefepime	Vancomycin-3rd generation cephalosporin	Vancomycin–piperacillin–tazobactam
A	1	Yeast	R	R	R	R
B	1	Yeast	R	R	R	R
C	1	*E. raffinosus*	R	S	S	S
	2	*E. raffinosus*	R	S	S	S
	3	ESBL-producing *E. coli*	R	R	R	S
D	1	ESBL-producing *E. cloacae*	R	R	R	R
	2	ESBL-producing *E. cloacae*	R	R	R	R
E	1	*E. coli*	S	S	S	R
F	1	*K. pneumoniae*	S	S	S	R
G	1	ESBL-producing *K. pneumoniae*	R	R	R	R
		OXA23-producing *A. baumannii*	R	R	R	R
H	1	ESBL-producing *E. cloacae*	R	R	R	R
	2	*P. aeruginosa*	S	S	R	S
I	1	*P. aeruginosa*	R	S	R	S
J	1	*P. aeruginosa*	S	S	R	S
K	1	*B. thetaiotaomicron*	R	R	R	S

*R* resistant; *S* susceptible

**Fig. 1:**
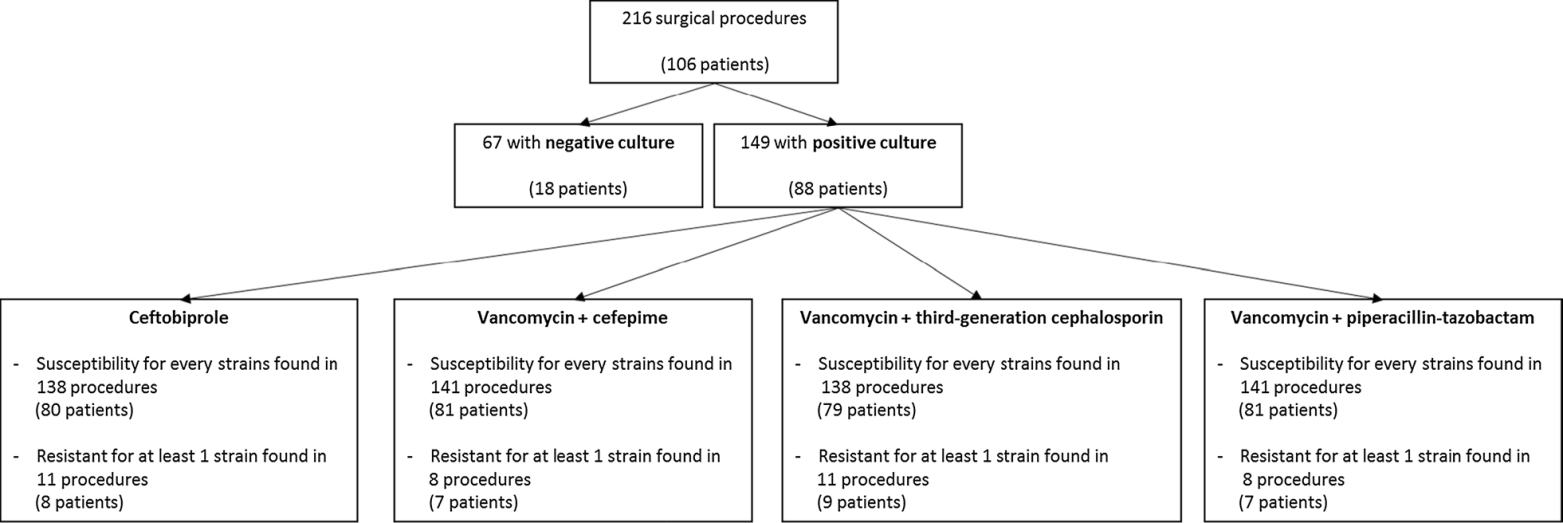
Antimicrobial susceptibility for the 216 surgical procedures

Finally, over the 216 reported surgical procedures, microorganisms were cultured for 149 of them. Based on antimicrobial susceptibility testing, ceftobiprole could have been used with success in 138 cases among these 149 interventions, versus vancomycin plus cefepime in 141 cases (p = 0.64), vancomycin plus a third-generation cephalosporin in 138 cases (p = 1) and vancomycin plus piperacillin–tazobactam in 141 cases (p = 0.64). Empirical use of ceftobiprole would have been ineffective in 8 patients (patients A, B, C, D, G, H, I, K) (Table 3; Fig. 1).

## Discussion

Ceftobiprole is currently not approved for the treatment of PJIs but its broad spectrum of activity including most of the bacteria responsible for these infections makes it an attractive candidate for empirical antibiotic therapy following surgical septic revision of PJIs. Our results suggest that ceftobiprole would have been effective on 92.6% of surgical procedures. Our epidemiology being like that found in the literature [4], ceftobiprole is non inferior compared to the three associations evaluated: vancomycin plus cefepime, a third-generation cephalosporin or piperacillin–tazobactam, in terms of microbial susceptibility. The good correlation between in vitro and in vivo activity of ceftobiprole has been confirmed [5] as well as the low potential for emergence of resistance under treatment [6].

Ceftobiprole is active against most of Gram-positive cocci, including staphylococci, which are the most prevalent bacteria identified in our patients, accounting for 74% of the microorganisms cultured). All are susceptible in vitro to ceftobiprole with MIC_50/90_ of 0.5/1 and one strain of CoNS with a MIC of 4 mg/L. These results are like those obtained by Isnard et al. [7] in a study performed on 100 *S. aureus* strains and 100 CoNS strains cultured from PJIs: all but 2 *S. aureus* strains (with MIC of 4 mg/L) had MIC lower than 2 mg/L. In another series of 33 strains of *S. epidermidis*, including one-third multiresistant strains, Hellmark et al. [8] found MIC_50_ and MIC_90_ values of 0.5 and 1.5 mg/L respectively.

In our study, 53.1% of CoNS and 6.6% of *S.* *aureus* were methicillin-resistant strains and all were susceptible to ceftobiprole. In the literature, Rouse et al. studied the activity of ceftobiprole against MRSA (31 isolates) and CoNS (65 isolates) from bone and joint infections: all strains had MIC ≤ 2 mg/L (except for one CoNS with MIC value of 4 mg/L) [9]. Our study also included 20.4% of teicoplanin-resistant CoNS, all remaining susceptible to ceftobiprole; these results are consistent with those obtained by Henriksen et al. [10] on 136 teicoplanin-resistant bacteremia CoNS strains (representing 20.9% of their 650 bacteremia CoNS strains). Concerning vancomycin-resistant strains, Farrell et al. [11] demonstrated a potential high activity of ceftobiprole against 44 VISA and hVISA strains (MIC_90_ of 2 mg/L) and against 10 VRSA strains (MIC_90_ of 1 mg/L).

In addition, the bactericidal activity of ceftobiprole against Staphylococci has been proved in several studies: Isnard et al. [7] showed a rapid killing effect, with a significant and sustained decrease of the inoculum after a 24 h incubation period at concentration of 4 * MIC while a phenomenon of re-growth was observed with vancomycin. Rouse et al. demonstrated it against 31 methicillin-resistant *S. aureus* and 65 CoNS isolated from bone and joint infections (all strains had MIC and MBC values ≤ 2 µg/mL, except for one CoNS with MIC and MBC values of 4 and 8 µg/mL) while vancomycin lacked bactericidal activity against 2 MRSA [9]. In vivo studies were also performed with ceftobiprole. For methicillin-susceptible strains, vancomycin is known to have a lesser efficiency against methicillin-susceptible strains compared with beta-lactams and ceftobiprole was superior to ceftriaxone and vancomycin in a mouse experimental septicemia model [5]. In vivo activity of ceftobiprole was also shown in a MRSA endocarditis model in rabbits [12] and in VISA infection models [5].

PJI may also be caused by streptococci, and Gram-negative bacilli on which ceftobiprole maintains the activity of extended-spectrum cephalosporins. In our study including 20 Enterobacteriales strains, a resistance to ceftobiprole was detected only for the 4 ESBL-producing strains, which were also resistant to cefepime and third-generation cephalosporins.

For anaerobic bacteria, it has a similar spectrum as amoxicillin plus clavulanic acid. It is active against *P. aeruginosa* strains, mostly in ceftazidime-susceptible strains but has a poor activity on *A. baumannii* [13, 14].

The main insufficiency of ceftobiprole in the setting of PEAT for PJIs are (i) ampicillin-resistant strains of *E. faecium* (on which vancomycin may be effective), (ii) strains of *B. fragilis* group species, and (iii) ESBL-producing Enterobacteriales (on which only piperacillin–tazobactam may be effective). These strains are not frequently found as causative agents of PJI and infections with ESBL-producing Enterobacteriales may be suspected on the basis of the patients’ medical history. Thus, and as for any antibiotic therapy, bacteriological history of the patient and microbial ecology of the hospital must be considered when choosing the PEAT.

Our study presents some limitations. First, one-third of the total specimens remained sterile. This may seem important, but we included surgical interventions in patients previously infected in order to control the sterility of the specimens. Furthertheless, it does not evaluate activity on microorganisms that grow in biofilms, which cannot be evaluated routinely. Biofilm impairs the activity of most antibiotics by reducing their accessibility to bacteria. Moreover, bacteria in biofilms are for some of them in a stationary phase [4] which results in an increased resistance to most antibiotics, especially those with a growth-dependent mode of action such as ceftobiprole. Nevertheless, an in vitro study performed by Abbanat et al. [15] showed a CFU decrease > 2log_10_ on immature and mature staphylococcal biofilms by ceftobiprole which seems to be less affected by biofilm age than vancomycin. Their results are consistent with in vivo results obtained in endocarditis infection model [12]. For treatment of PJI, antibiotics used must diffuse in the bone tissue. Yin et al. [16] studied pharmacokinetics and distribution into bone tissue of ceftobiprole in a rabbit model of osteomyelitis, pointing out promising results with bacterial titers in infected tibiae below the level of detection after treatment with ceftobiprole (versus 73% of the infected tibiae treated by vancomycin) and mean titers of the molecule 3 to 5 times higher in infected bones than in uninfected bones. Clinical data, however, are lacking. It may be useful to use a single molecule rather than an association in terms of adverse effects of the therapy.

Our results suggest that ceftobiprole used as PEAT for PJIs shows similar spectrum of activity as the associations vancomycin plus a beta-lactam currently recommended and could be an attractive alternative to the combination antibiotic therapy currently proposed.

## Data Availability

Not applicable to data. All information utilized could be found using the references provided in body of the manuscript.
